# Factors Associated with Burden in Caregivers of Patients with End-Stage Kidney Disease (A Systematic Review)

**DOI:** 10.3390/healthcare9091212

**Published:** 2021-09-14

**Authors:** Bushra Alshammari, Helen Noble, Helen McAneney, Farhan Alshammari, Peter O’Halloran

**Affiliations:** 1 Medical Biology Centre, School of Nursing and Midwifery, Queen’s University Belfast, 97 Lisburn Rd, Belfast BT9 7BL, UK; helen.noble@qub.ac.uk (H.N.); p.ohalloran@qub.ac.uk (P.O.); 2 College of Nursing, University of Hail, Hail 2440, Saudi Arabia; 3 UCD Centre for Interdisciplinary Research, Education and Innovation in Health Systems, School of Nursing, Midwifery and Health Systems, University College Dublin, Dublin, Ireland; helen.mcaneney@ucd.ie; 4 Department of Pharmaceutics, College of Pharmacy, University of Hail, Hail 2440, Saudi Arabia; frh.alshammari@uoh.edu.sa

**Keywords:** caregiver burden, caregiver strain, caregiver stress, end-stage kidney disease, renal failure, systematic review

## Abstract

Background: Caring for a patient with end-stage kidney disease (ESKD) is highly stressful and can impact negatively on the physical and psychological well-being of caregivers. To accurately assess caregiver burden (CB), health care providers (HCPs) need to identify characteristics associated with an increase in CB. Aim: The aim of this review is to explore CB in caregivers of adult patients with ESKD and to identify characteristics associated with any increase in CB. Method: A comprehensive literature search was completed using five electronic databases. Medline, Embase, CINHAL, PsycINFO, and Scopus. The Joanna Briggs Institute checklist (JBI) was used to quality appraise full text papers included in the review. No time limit for the date of publication of studies was employed, to enable the inclusion of more extensive literature. Results: A total of 38 relevant studies from 18 countries were identified and included in the review. A variety of patient and caregiver factors can impact positively or negatively on CB, including socio-demographic factors of patients and caregivers, disease-related factors, situational and relational factors, environmental factors, and psychological factors. Conclusion: This review provides awareness to HCPs of the important factors associated with CB, when assessing or targeting interventions for caregivers experiencing burden.

## 1. Introduction

The population with chronic kidney disease (CKD) is increasing due to the growing prevalence of hypertension, diabetes, and obesity. Globally, CKD has a prevalence of between 11 to 13.4% in the general population, which makes it one of the most common worldwide diseases [1]. When CKD is not properly managed, it can progress to end-stage kidney disease (ESKD), which is the final, permanent stage of CKD, where kidneys are no longer able to function properly to meet the patient’s needs. Patients with ESKD require lifelong replacement of kidney function by dialysis or transplantation to survive, and many are unable to meet self-care needs and require support from family or friends who are caregivers. The major responsibilities undertaken by caregivers include managing patients’ medical treatments, dietary requirements, and clinic and dialysis appointments [2]. Living with an individual in the advanced stages of CKD, and being the main caregiver is associated with challenges such as depression, anxiety, and increased use of medication for caregivers [3]. Caregiver burden (CB) can be defined as the extent to which caregivers perceive their emotional or physical health, social life, and financial status as deteriorating because of caring for their relative [4].

In the ESKD population, many factors are related to CB, such as the demographic characteristics of both caregivers and care recipients, ethnicity, comorbidity status, cohabiting with the patient, relationship with the patient, and duration of caring [3,5,6]. A recent mixed-methods systematic review explored the experiences of family members and friends who provided support for adults receiving haemodialysis (HD) or peritoneal dialysis (PD) [2]. This review considered the association between caregiver demographics and feelings of burden but did not address other factors associated with CB, such as patient characteristics, relationship duration, comorbidity, culture, and type of dialysis received by the care recipients. Consequently, a comprehensive and comparative overview is necessary to inform researchers and health care professionals of the impact these important factors can have on caregiver experiences. Understanding all of the factors is necessary to determine how health care professionals can provide appropriate and effective assistance to improve the quality of caregiving and reduce CB [7]. The aim of this review was to analyze studies that quantitatively measured the level CB among caregivers of adult patients with ESKD and to summarize the factors associated with CB.

## 2. Materials and Methods

### 2.1. Search Strategy

The Preferred Reporting Items for Systematic Reviews and Meta-Analysis (PRISMA) checklist was used to guide the reporting of the systematic reviews and to improve the quality and transparency of the data included [8]. The search was conducted in February 2019 and updated in December 2020 with assistance from the university librarian and was not limited to a specific time period. Included studies were identified following a search of electronic databases; Medline (1982–2020), Embase (1974–2020), CINAHL (1982–2020), PsycINFO (1809–2020), and Scopus (1985–2020). The following search terms were used: (Dialysis OR h*emodialysis OR Peritoneal Dialysis OR renal failure OR Renal Insufficiency OR kidney failure OR Kidney Diseases) AND (carer* OR caregiver*) AND (Burden OR strain). Terms were searched in the English language. The search also included reference lists contained within review studies and other relevant published reviews. Databases were searched individually and a combined search was subsequently completed. ProQuest RefWorks and Endnote were used to manage references electronically and to remove duplicate studies. The Boolean ‘OR’ featured aided in broadening the search, while the ‘AND’ helped narrow the search to identify relevant studies in each database.

### 2.2. Eligibility Criteria

1. Studies including informal caregivers of adult patients with ESKD, either undergoing dialysis or receiving supportive/palliative care.
2. Studies measuring CB and the factors associated positively or negatively with CB. In all studies included in this review, caregiver burden was defined as the extent to which caregivers perceive their emotional or physical health, social life, and financial status as becoming worse because of caring for their relative [4] measured by burden-specific instruments such as the Zarit Burden Interview (ZBI).
3. Studies examining informal caregiving of patients with renal transplant (RT) were excluded.
4. Studies exploring informal caregiving of patients with early stages of CKD were excluded.
5. Studies measuring CB in informal caregivers of patients with a range of chronic illnesses, which did not differentiate the burden experienced by informal caregivers of individuals with renal disease, were excluded.
6. Studies published in English were included.
7. No time limit was employed to exclude studies, to help maximise the number of studies included.
8. Studies assessing the effectiveness of interventions in CB were excluded.
9. Primary research studies were included.
10. Reviews, conference abstracts, dissertations, editorials, or researcher opinions were excluded.

### 2.3. Selection of Studies 

**Figure 1:**
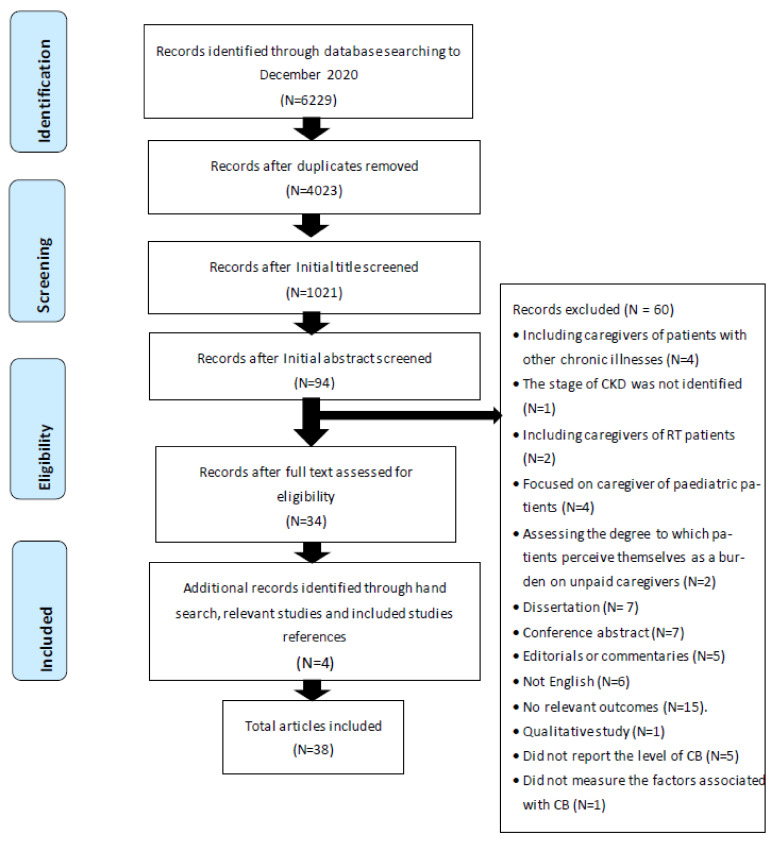
PRISMA flowchart diagram.

After duplicate studies were removed, 4023 titles and abstracts from all databases were independently screened for eligibility by three authors: B.A., H.N., and P.O. After initial title and abstract screening, 94 full text articles were read and the content discussed with the review team, to assess suitability for inclusion and to resolve any disagreements concerning inclusion or exclusion. A total of 60 studies were excluded as they did not meet the eligibility criteria. Four additional studies were added, as they had been identified in other relevant reviews, dissertations, or reference lists of included studies [9,10,11,12]. Therefore, 38 articles are included in this review, as illustrated in flow diagram which is outlined by the Preferred Reporting Items for Systematic Reviews Figure 1 [8].

### 2.4. Data Extraction 

The following data were extracted from each study: identification data (author’s name, year of publication and setting), methodological data (study design, number of participants, aim and method of measurement), and key findings (CB levels and factors associated with burden). Data extraction from all the included studies was performed by BA, PO and FA, and results were compared for consistency. Any discrepancies were resolved by discussion and reappraisal.

### 2.5. Quality Assessment Tool

We assessed the quality of the included studies to evaluate their methodological rigor and strength of the evidence they provide. The Joanna Briggs Institute (JBI) quality assessment tool was used to evaluate all studies included in the review [13]. This tool is designed to be used in systematic reviews to assess the methodological quality of a study and to determine the extent to which a study has addressed the possibility of bias in its design, conduct, and analysis. This tool employs a range of criteria to assess a variety of research study designs. The JBI Critical Appraisal Checklist for Analytical Cross-Sectional Studies was used to screen the final list of cross-sectional studies in domains including clearance of inclusion and exclusion criteria, description of the sample, location of the study, validity and reliability of the outcome measures, appropriateness of statistical analysis, identification of confounding variables, and how these have been considered. Each question can be answered as “yes”, “no”, “unclear”, or “NA” (not applicable).

The quality score was calculated by aggregating the number of ““yes” responses to all individual criteria, with a maximum score of 8. A score less than 3 indicates a low-quality study, a score between 3 to 5 a study of moderate quality, and a score of 5 or higher is a study of high quality. Two authors (BA and PO) performed the quality assessment of all included studies, which was subsequently reviewed and discussed by all authors. The authors assigned specific ratings of high, moderate, or low quality to all studies included. Some of the included studies failed to report the standard criteria used to measurement CB, and did not identify the setting in which the study was completed; however, all studies achieved an overall moderate to high quality score with a low risk of bias and none were excluded on this basis. (Table A1 in Appendix A).

### 2.6. Data Analysis, Synthesis, and Presentation

A descriptive narrative synthesis of the findings of each study was performed [14]. This decision was made because narrative synthesis provides a broad overview of relevant information, through a textual approach, which is appropriate when studies are too heterogeneous, to allow for a quantitative summary [15]. We assessed the studies to be too heterogeneous due to inconsistencies in methodologies, sample characteristics, and the instruments used to measure CB. The initial synthesis involved searching of studies, listing, and presenting the findings in tables. Then, the findings were sorted into five thematic categories based on their common characteristic with the frequency of studies within each theme presented. Subsequently, the included studies were summarised in a narrative synthesis, which was performed by one author and discussed and agreed by the review members.

## 3. Results

### 3.1. Characteristics of Included Studies 

The characteristics of the final studies are shown in Table 1. The search identified 38 studies that met the eligibility criteria. Of the included studies, 35 used a cross sectional design [5,9,10,11,12,16,17,18,19,20,21,22,23,24,25,26,27,28,29,30,31,32,33,34,35,36,37,38,39,40,41,42,43,44,45] and three provided cross sectional data within other designs; one used a quasi-experimental design [46], one used a longitudinal design [47], and one a mixed method design [6]. Studies included in the review were completed in a variety of countries, including: seven in Turkey [11,22,23,24,27,33,46], four in the USA [5,18,29,44], four in Iran [31,34,37,39], two in Brazil [25,26], two in Japan [34,41,43], two in Saudi Arabia [9,21], three in China [12,42,45], two in Nigeria [6,17], two in Jordan [16,19], two in India [32,35]; and a single study in Nepal [10], Canada [38], Pakistan [40], Greece [36], Spain [20], Singapore [47], Vietnam [30], and Indonesia [28]. CB was assessed in a variety of individuals with ESKD, including: HD in 21 studies, PD in four studies, both HD and PD in four studies, PD and RT in one study, HD and RT in one study; and PD, HD, and RT in three studies. Three studies investigated ESKD without specifying if subjects are dialysis dependent or not, and a single study included caregivers of both dialysis-dependent and non-dependent patients. All the reviewed studies were published between 1997 and 2020, see Table 1.

The most common tools used to measure CB was the ZBI [48] in 29 studies, Caregiver Burden Scale (CBS) [49] in four studies, Novak and Guest Care Burden Questionnaire in three studies [50], with single studies using the Oberst Caregiving Burden Scale (OCBS) [51], Caregiving Stress Appraisal (CSA) [52], and the Caregiver Strain Index (CSI) [53].

### 3.2. Level of CB

In this review, 38 studies investigated the level of CB in advanced stages of CKD patients and reported contrasting findings. Three studies reported high levels of burden [5,6,16]. The majority of the included studies reported moderate to severe levels of burden in caregivers of patients undergoing HD [9,10,18,20,21,27,30,32,34,37,39,46]. Several studies reported moderate CB [11,19,25,26,31,36]. Three studies reported moderate CB in the caregiver dialysis group and low CB in caregivers of RT patients [22,23,35]. Mild to moderate burden was reported in caregivers of patients receiving HD [12,17,33,40,42,43,45,47] and patients receiving PD [27]. One study reported mild to moderate CB in caregivers of patients receiving PD, HD, and RT [24]. Several studies reported mild to no burden in caregivers of patients receiving HD [28,38,41] and PD [44]. Low burden was also reported in one study of dialysis dependent patients, without specifying whether patients were HD dependent or PD dependent [29].

### 3.3. Factors Associated with CB

The factors associated with CB were sorted into five thematic categories based on their common characteristic, with the frequency of studies within each theme presented, see Table 2. The content of each category is described as follows:

1. Caregiver’s and patient’s socio-demographical factors including age, gender, ethnicity, marital status, education, employment, income, ethnicity and race, and religion/spirituality.
2. Disease-related factors including treatment modality, frequency of weekly dialysis sessions, duration and frequency of dialysis, duration of illness, comorbidity, level of patient’s dependency, and quality of Life (QoL).
3. Situational and relational factors including cohabiting status, relationship to the patient, the duration of caregiving, contact time per week, duration of relationship with patients, number of children, smoking and alcohol consumption, and caregiver performing exercise.
4. Environmental factors including social support.
5. Psychological factors including depression and anxiety.

#### 3.3.1. Caregiver and Patient Socio-Demographical Factors

##### Age of Caregivers and Patients, and Perceived CB

Twenty-seven studies of the thirty-eight studies reviewed explored the age of caregivers [5,9,10,11,12,16,19,21,22,24,26,27,28,29,30,31,32,33,34,36,37,39,45,46] and patients as a predictor of CB [9,16,19,20,24,25,31,34,37,39,41,45]. Four studies highlighted a negative correlation between CB and caregiver age [9,21,25,46]. In contrast, four studies reported that older caregivers have greater difficulty coping and experience higher levels of burden than younger caregivers [5,10,12,16,28,30,31,37,39]. Rodrigue et al. [5] reported higher CB with increased caregiver age, although this study did not find a statistically significant correlation and suggested that further research is required, using a larger sample population to determine if a relationship exists.

Caregivers of older care recipients had a significantly higher level of CB than those who provide care to younger care recipients [9,20,39,41], while a single study reported high scores in one of the CB components (environmental component) in caregivers of younger patients [25]. The remaining studies reported that caregiver age did not influence feelings of burden in caregivers [11,19,24,26,27,29,32,34,45] or patient age [16,19,24,26,31,34,37,45].

##### Gender and CB

Gender has frequently been investigated as a factor in CB. Twenty-three studies explored the gender of caregivers [5,6,11,16,17,19,22,24,25,26,27,28,31,32,33,34,36,39,42,44,45,46] and patients [16,19,24,25,26,31,34,37,39,42,44,45] as a contribution to CB. Many studies reported that female caregivers experienced greater feelings of burden than male caregivers [5,6,11,17,26,32,42,45,46]. However, two studies reported that this association did not reach a statistically significant level [5,6]. Two studies suggested that providing care to male patients increases the level of reported CB, regardless of the gender of caregivers [26,34]. A number of studies have reported that there is no evidence of a relationship between CB and caregiver gender [16,19,22,24,25,27,28,31,33,34,36,39,44] and this was also the case for patient gender in several studies [16,19,24,25,31,37,39,42,44,45].

##### Marital Status and CB

Twelve studies examined the relationship between caregiver marital status (being single, married, widow or divorced) and reported CB [6,10,11,16,24,30,31,33,34,37,39,46]. Two studies reported that single caregivers experienced higher levels of burden when caring for ESKD patients than married caregivers [34,46]. These findings are discussed by Mashayekhi et al. [34], who reported that being single played a significant role in some CB components, including disappointment, lack of freedom, financial difficulties, and feelings that life is unfair. However, younger caregivers reported being confident in their ability to provide care and cope with patient problems [34]. Shakya et al. [10] reported that widowed caregivers reported higher CB levels than those who were married, with the lowest CB in single caregivers.

The majority of studies reported that there was no relationship between CB and the marital status of caregivers [6,11,16,24,31,33,37,39]. No association between CB and the patient’s marital status was found [16,24,34,37,39]. One study investigated marital satisfaction levels reported by caregivers, with no relationship identified [34].

##### Education, Income and Employment 

A number of additional socio-demographic factors have been explored to determine if they contribute to CB. Twenty-two studies examined the relationship between CB and caregiver’s educational attainment [6,9,10,11,12,16,19,21,22,24,26,27,28,31,33,34,36,37,39,42,45,46]. Fifteen studies reported no correlation, while six studies reported a negative association between CB and the educational attainment of caregivers [9,10,12,21,31,37]. In contrast, a single study reported increased CB in caregivers with higher educational attainment [46]. However, this study reported that caregivers who receive higher levels of education in how to support the care recipients experience a decrease in the burden of caregiving [46]. Similarly, caregivers with adequate health literacy reported lower levels of CB [16].

The association between CB and patient educational attainment has also been investigated [9,16,19,24,26,34,37,42]. Three studies found that lower levels of educational attainment contributed to higher levels of CB [9,26,37], while the remaining studies reported no relationship [16,19,24,34,42].

Twelve studies examined caregiver income as a predictor of CB [11,12,16,22,27,28,30,31,34,42,45,46]. Lower caregiver income was found to be a factor in higher CB [11,28,34,42], while no association was reported in the remaining studies. Additionally, five studies explored patient’s income as a contributor to CB, two of these studies reported that low patient income was associated with higher CB [12,28], while the other studies reported no association [16,26,39].

Thirteen studies examined the association between CB and the employment status of caregivers, including employed, unemployed, retired, full-time, and part-time work [6,11,12,16,24,26,30,31,34,36,37,44,46]. Two studies suggested that feelings of burden were greater in unemployed caregivers [12,37]. However, Cagan et al. [11] reported that higher CB was more evident in caregivers who were employed. Four studies examined the employment status of both caregivers and patients and found no correlation with CB [16,24,26,34], while one study reported no association between employment status of caregivers and CB [30].

##### Ethnicity, Race and Religion 

A small number of studies have investigated ethnicity and the race of caregivers in patients with ESKD and reported no correlation with perceived CB [6,44]. Only one study examined caregivers who identified with specific religious groups (Islam and Christianity). No correlation with CB was found [6]. The spiritual well-being of caregivers was negatively associated with CB [37].

#### 3.3.2. Disease-Related Factors

##### Comorbidity, Duration of Illness, Patient’s Dependency, Health Status and QoL

Several studies investigated caregiver comorbidity as a CB predictor [11,16,20,26,30,43,46]. Five studies reported that the burden is greater in caregivers who experience comorbid conditions [16,20,30,43,46], while two studies reported no correlation [11,26]. In relation to patient co-morbidity, five studies reported a significant positive correlation between this and CB [12,20,26,31,45], whereas one study reported no association [16].

CB is likely to increase when a patient’s physical health deteriorates and patient functional independence declines [5,31,37,40,42,45]. In addition, CB appears to increase when caregivers also experience poor physical and mental health [18,44]. Two studies investigated the duration of illness and reported no relationship between illness duration in patients with ESKD and CB [9,21]. Two studies explored the relationship between CB and HRQoL in caregivers and reported a negative correlation to CB [5,41]. Three studies reported a negative association between CB and QoL [31,44,45], and one study reported no association [17].

##### Duration and Frequency of Dialysis, and Treatment Modality

The duration of dialysis in care recipients has been explored and found to be positively correlated with the level of CB [11,24,40,45]. A positive association between the frequency of dialysis and CB also was found [37]. However, other studies reported no association between CB and dialysis duration [10,16,26,31,34,37,39] or dialysis frequency [10,11,34].

Several studies investigated treatment modality and whether patients were dialysis dependent or not, in relation to reported CB [5,20,21,22,23,24,25,27,35,36,37,44]. CB scores were found to be higher in caregivers of patients receiving HD compared with caregivers of patients receiving PD [24,27] and RT groups [23,24,35], as well as for patients who were not yet receiving dialysis [5]. Caregivers of patients with RT reported lower CB than patients receiving peritoneal dialysis [22]. The CB level was lower in caregivers whose patients had a kidney transplant history than the ones who did not [37]. On the other hand, several studies reported that CB levels did not differ by dialysis dependency (dialysis dependent or not) [36,44], type (PD, HD, home or in-centre HD) [20,21,25,44] or before and after RT [5].

#### 3.3.3. Situational and Relational Factors 

##### Caregiver relationship to patients, cohabiting arrangements, place of residency, duration of caregiving and duration of relationship between patients and caregivers

Several studies examined the effect of caregiver relationships in patients with CB [6,10,11,12,16,19,24,26,30,33,34,37,39,42,43,44,45,46]. Seven studies reported that the nature of the caregiver relationship with the patient influences the levels of CB [10,12,26,33,39,43,46].

In five studies, caregivers who were spouses of patients were found to have a comparatively high level of CB compared to those with other types of relationships [10,12,26,39,43]. Three of them have reported that high levels of CB can also be observed in parents of patients [10,12,43].

On the other hand, two studies of caregivers of patients receiving HD in Turkey reported that spouse caregivers have less burden compared to other family relatives such as sons, daughters, siblings, grandmothers and grandfathers [33], daughters, daughters-in-law, and siblings [46]. However, a number of studies suggested that the nature of the relationship with the patient did not predict CB [6,11,19,24,34,44]. A single study reported a positive correlation between the duration of the relationship between caregivers and care recipients and caregiving benefits [5].

Only one study highlighted that caregivers living with patients, irrespective of the relationship, experienced greater CB than relatives who live in a separate residence [19,45], while four studies reported no effect [6,11,24,26,42]. The place of residency (either city or rural area) has been investigated in patients receiving in-centre HD and found that those living in a city location had lower CB scores than those in rural areas [33,46]. However, one study reported no association between the place of residence and CB [11].

The caregiving duration was examined as a predictor of CB in ten studies [11,16,19,20,26,27,30,32,43,47]. Six studies reported a positive correlation between the duration of caregiving and CB [11,26,30,32,43,47], while remaining studies reported no relationship.

The time spent providing care (hours/day) has also been investigated. Longer daily hours of caregiving were found to be positively correlated to the level of CB [12,20,30,32,40,45], but five studies found no correlation [6,19,26,28,42].

A single study investigated a variety of variables including: the number of children, smoking habits, and alcohol consumption, and reported that caregivers having three or more children have higher CB, with no changes in CB associated with smoking and alcohol consumption [11]. One study identified a positive association between CB and the number of medications used by patients [20]. One study found a lower CB in the caregivers who exercised for more than one hour a week [45].

#### 3.3.4. Environmental Factors

Caregivers who seek social support from family and friends experience less CB than caregivers without strong support networks [10,19,20,42]. Social support diminished the impact of emotional CB and stress by providing a solution to the problem, by providing distraction from the problem or by facilitating healthy behaviours needed [10,19,20]. The studies did not report how social support can reduce the physical burden of care.

#### 3.3.5. Psychological Factors

All studies that investigated the relationship between caregiver depression and anxiety and CB reported that higher levels of psychological symptoms are correlated with increased CB [10,17,24,36,38,43]. Caregivers of patients who experience depressive symptoms reported higher levels of CB [42].

## 4. Discussion

This review synthesised the current evidence related to CB and the factors associated with CB in patients with ESKD. Informal caregiving research has largely focused on patients with cancer and mental illness, including dementia and Alzheimer’s disease, with limited research on caregiving in patients with renal disease. This systematic review includes research from a range of countries. Cultural values and social patterns in these countries are presented in the review, which allows judicious generalisation of the findings to relevant cultural contexts. However, there are several limitations of this review that should be acknowledged. Some potentially relevant studies were unavailable in English [54,55,56,57,58,59]. Additionally, the majority of studies included in the review used a convenience sample, which means study participants may not be representative of their populations. Moreover, due to the range of different instruments used to measure CB, a variety of aspects of CB have been reported. Each instrument used different components to measure CB, for example, the CSA comprises two components: social constraints and physical exhaustion [52]. The ZBI includes items related to consequences of caregiving, guilt or self-criticism, patient’s dependence, frustration/embarrassment or anger, emotional reactions and psychological burden, personal strain, and role strain [60], while the CBS contains isolation, disappointment, and emotional involvement items [49]. These differences in the measurement of CB are due to the lack of a conceptualisation or agreed definition of CB in the literature [7,61], which may contribute to differences in study findings [62].

Caregivers of patients with ESKD experience a significant burden, regardless of country of residence. Burden levels in caregivers are likely to be regulated by a wide range of factors. These factors include socio-demographic characteristics of caregivers and patients, disease-related factors, caregiving-related factors, environmental factors, and psychological factors. However, some factors are relatively consistent across studies, whilst for others, findings are inconsistent or inconclusive.

### 4.1. Consistency between Studies 

The studies included in this systematic review consistently report that gender, caregiver and patient income, time providing daily care, duration of caregiving, the relationship to patients, and cohabiting arrangements are associated with increased CB. Being a female caregiver is frequently reported as a contributor to higher CB, in patients with ESKD. This finding was consistent with the findings of a systematic review conducted on caregivers of patients with dementia [7]. Several factors explain this finding. For example, gender differences and burden may be due to gender roles, with women still largely regarded as the primary caregiver in social and informal care situations [63]. Therefore, women may self-impose the duty of caregiving more readily than their male counterparts. Alternatively, women may more readily voice their experiences of caregiving versus men, which may be aligned to gender roles that typically result in women being more expressive and vocal about their emotions [64]. Accordingly, women are more likely to express negative feelings than men [65,66]. In contrast, in Middle Eastern countries, men are less willing to complain and express weakness due to cultural norms that reinforce that men are strong and able to tolerate stress [67]. Consequently, differences between men and women may in part reflect differences in their willingness to report CB.

This review suggests that individuals within lower socio-economic populations (the unemployed or on low incomes) may experience higher CB. The explanation for this finding is that caregivers with limited income may have a lack of adequate facilities to meet patient requirements, limited access to suitable care and medication, a lack of transportation, difficulty accessing medical facilities, and limited contact with social support organisations [26]. Caregivers of patients with dementia in lower socio-economic groups have also been found to experience higher CB [68].

A longer duration of caregiving and spending a longer time providing daily care were found to be associated with increased CB. These findings may be due to the poor health status of patients that require more time to care for. This is consistent with the findings of Serrano-Aguilar et al. [69] and Conde-Sala et al. [70], who explained that when patients have lower levels of wellbeing, caregivers would be expected to assist in providing further hours of care. Spending hours in caregiving responsibilities may lead to limitations in carrying out daily personal duties [71], and restrictions in participating in social activities [72]. Furthermore, a longer time spent on caregiving might be due to the difficulty of caregiving tasks associated with CB [30].

Living with patients at the same residence was found to increase CB. The same results were also stated by Raccichini et al. [73] and Viñas-Diez et al. [74], who explained that living with the patients, most likely to be spouses, involved constant patient care leading to a greater physical, emotional, and social burden, which increase over time.

Living in a peripheral district was associated with increased CB. Notably, studies reported that caregivers living in rural areas may report higher levels of CB, which may be due to the increased probability that these patients require in-centre dialysis treatment, or the additional physical and financial burden of travelling to dialysis centres.

Social support was found to help reduce CB [10,19,20,42]. According to Alnazly [19], in the Kingdom of Jordan, spouses, children, and siblings are all involved physically and emotionally in caregiving, which helps to minimise feelings of burden in primary caregivers. Additional studies are required to identify the association between the numbers of individuals involved in providing care and the level of CB.

In this systematic review, studies reported that disease-related factors are consistently associated with increased CB. Burden for caregivers of those with receiving HD was significantly higher than for caregivers of patients within PD and RT groups. These results can be explained by the essential differences between treatment modalities. Patients undergoing HD typically attend a dialysis centre three times a week and spend a minimum of three hours per session. Unlike patients on HD, patients receiving PD experienced fewer symptoms, less pain, and are better able to maintain their personal lives and social interaction actively [75], which decreases the amount of support required from caregivers and therefore reduces CB.

Higher numbers of comorbidities in patients and caregivers were associated with higher levels of CB. These findings are similar to those reported by informal caregivers of patients with dementia [76]. Caregiving responsibilities and the amount of time needed to provide care were likely to increase when a patient’s physical health deteriorated, reducing their ability to perform daily activities and increasing their functional dependence. Caregivers may feel overwhelmed with managing the complex needs of patients with comorbidities, while at the same time they are dealing with the presence of CKD and dialysis treatment leading to increase CB. Furthermore, this review revealed that the number of medications used by patients was associated with CB [20]. Patients may take medications due to the presence of other comorbidities that increase CB.

Psychological symptoms such as depression and anxiety were significant contributors to CB. These findings support earlier research with caregivers of those with dementia, which reported that psychological symptoms, experienced by caregivers, were the main factors that contribute to CB [7].

Zhang et al. [45] suggested that performing exercise for more than one hour a week can reduce CB. Limited studies focus on the caregivers doing exercise to relieve CB. One study focused on the effectiveness of the involvement of care recipients in intradialytic exercise and found that it successfully reduced CB [77].

### 4.2. Inconsistent Findings

A number of socio-demographic factors reported inconsistent findings in relation to CB. The age of caregivers and patients was found to affect CB both negatively and positively. Younger caregivers may be more vulnerable to the challenges imposed when caregiving, and this may result in greater social isolation and financial insecurity [78], and reduced problem-solving skills [46]. On the other hand, older caregivers may be limited physically and mentally, which may influence their caregiving abilities [79]. Both explanations are credible, and these differences may be due to the multinational research included in the review, which considers this issue in a diverse range of cultures. It is clear that culture shapes caregiving attitudes; this conclusion is supported by a number of authors [80,81], but only two studies explored the ethnicity, race, and religion of caregivers, and these found no effect on CB. Further research is required to examine cultural differences and the effect on CB.

Caregivers with low education levels or caring of patients with low education had higher CB scores, with the exception of one study, which reported contradictory findings with more educated caregivers reporting higher CB [46]. The authors of this study suggest that caregivers with higher educational attainment may commit to other responsibilities and have higher expectations for their lives rather than dedicating themselves totally to caring. Additionally, caregivers with adequate health literacy [16] and receive higher levels of education in how to support the care recipients experience less CB [46]. Caregivers who have the ability to gain access to and understand relevant information may use this to help maintain and promote patients’ health, and so experience lower CB. Caregivers with adequate health knowledge are prepared more to support their patients and having less concern about performing tasks such as dealing with disease symptoms, preventing infection, and assisting with side effects [82]. This review highlights that single caregivers experience greater feelings of burden, in contrast to one study, which suggested that single caregivers reported the lowest CB [10]. Burnley [83] argues that having a spouse (who is not the patient) who can provide support in times of stress can reduce feelings of burden. Married caregivers may receive support from partners, a resource that single caregivers cannot avail of [83]. The author suggests that marriage can be a source of solace and support and can help to reduce burden [83]. In this review, only one study reported that being married was a significant predictor of higher CB in patients with ESKD [30], which was similarly demonstrated in patients with dementia [84]. Marital status has been investigated extensively in CB literature, but marital satisfaction levels may be a key to positive caregiving outcomes, as more cooperative couples may engage in more adaptive behaviours and positive self-management [85]. In this review, only one study investigated marital satisfaction in relation to CB, and found no correlation, although this may be due to a small sample size [36]. Additional research is required to identify if any association exists between marital satisfaction and CB.

The study of caregiver relationship to patients reported some varied findings between studies. Five studies report that spouses experience higher, while two studies suggested that parent caregivers reported high CB. The findings in this review and in additional studies demonstrate that close family members are more likely to experience higher CB than other relatives or unrelated individuals [86,87]. This may be because family members are more emotionally involved in their duties as caregivers, and so feel obliged to take care of the family member, even when their personal well-being is compromised [87].

Spouses in particular may experience a greater sense of responsibility, which intensifies the emotional content of caregiving, leading to a greater burden [88]. The challenges facing the spouses who are being primary caregivers and the expectations from them are different from any other relative caregivers. The challenges included managing a drop in income, dealing with additional parenting responsibilities, and the lack of intimacy or reciprocity within the marital relationship [89]. On the other hand, two studies conducted in Turkey reported lower CB in spouses [33,46] and suggested that this is influenced by Turkish culture where spouses perceiving the caring role as a normal task rather than a burden [33]. It is clear from these studies that findings cannot be generalised to different contexts, in light of the differences in caregiver relationships across different regions. For example, more traditional values make it clear that daughters are the preferred caregivers in Middle Eastern countries [46], while the spouse is judged to be the most appropriate caregiver in Canada [38]. This suggests that there may be a disparity in CB in patients across a wide range of cultural settings.

## 5. Conclusions

Increased reported CB is associated with female sex; carer anxiety, depression, and ill-health; caring for patients receiving HD and with poorer health; spending longer time giving care; lower socio-economic status; and living a significant distance from a dialysis centre. A longer duration of relationship between caregiver and patient, increased marital satisfaction, and social support from other family members may provide protective effects. Understanding all the of the factors is necessary to determine how health care professionals can provide appropriate and effective assistance to improve the quality of caregiving and reduce CB. The current evidence suggests that the differences in the levels of CB between studies might be due to the influence of cultural variables, which need further investigation. Further research is needed to clarify the relationship between CB and caregiver age, educational level, marital and relationship status, together with the influence of cultural norms on the caregiver role and experience. Those supporting caregivers of patients with ESKD should recognise that most caregivers will experience at least moderate CB, and that this may increase as the patient’s condition deteriorates. Assessing CB should be a regular component of care for people with ESKD and caregivers with characteristics associated with increased CB may need targeted additional support. This is necessary to avoid early nursing home placement, reduce the adverse health outcomes for patients, and prevent the deterioration of caregivers’ health. HCPs should inform caregivers to consider seeking social support, performing exercise, and obtaining an adequate education in how to support the care recipients when experiencing burden.

## Figures and Tables

**Table 1:** Summary of the included studies.

Author/Year/Country	Aim	Design/Sample	Caregiver Burden Level/Measurement Tool	Factors Associated with CB
Abed et al. (2020) Jordan	To investigate the functional health literacy and CB among family caregivers for patients receiving HD	Cross sectional study of 88 caregivers of patients on HD	Severe CB level using ZBI	Positive relationship of caregivers’ age to CB but no with patient age, history of comorbidity of caregivers. Caregivers with adequate health literacy had less CB than caregivers with limited health literacy.
Adejumo et al. (2019) Nigeria	To measure burden, psychological well-being, and QOL of caregivers of patients with ESKD	Cross sectional study of 57 caregivers of patients on HD	Mild to moderate CB level using ZBI	CB of female caregivers and experience higher scores of anxiety and depression were significantly higher than male caregivers.
Affinito and Louie (2018) USA	To explore the association between CB and the levels of health of caregivers of HD patients	Cross sectional study of 89 caregivers of patients on HD	Mean of 29.38 out of a total score of 48 indicating moderate to severe CB using CSA	Caregivers with good level of health, and who viewed their caregiver role positively, experienced a lesser degree of CB.
Alnazly (2016) Jordan	To explore the burden and coping strategies of caregivers of patients receiving HD	Cross sectional study of 139 caregivers of patients on HD	Moderate degree of burden using OCBS	Living with the patient was the only variable positively correlated to CB. Caregivers’ and patients’ age, gender, education, employment status, relation to patient, years of caregiving, and caregiving hours per week were not significantly related to CB.
Alvarez-Ude et al. (2004) Spain	To evaluate the HRQoL and burden of family caregivers of chronic dialysis patients and analyse the factors associated with it	Cross sectional study of 221 patients/caregivers of patients on HD and PD	Moderate to severe burden using ZBI	CB was higher in caregivers with less social support, and poor physical and mental health. Age of patients, number of caregiving hours, number of comorbid conditions of patients and caregivers, number of medications of patients, correlated positively with CB. No differences in CB were found between caregivers of patients with HD and PD.
Al wakeel and Bayoumi (2016) Saudi Arabia	To compare the burden on family caregiver between HD and PD in Saudi Arabian population	Cross sectional study of 105 caregivers, 50 caregivers of patients receiving HD and 55 caregivers of patients receiving PD	Mean CB in patient receiving HD was 43.3 (21.7) and 49 (24.5) in caregivers of the PD group. Both reported moderate to severe burden using ZBI	No significant differences between CB in caregivers of patients receiving PD and HD. Caregiver’s age and education level correlated negatively to CB in the HD group. Age and level of education and dialysis duration were not correlated to CB in PD group.
Avsar et al. (2013) Turkey	To examine the relationship between caregivers of PD and RT patients with regard to sleep quality, anxiety, depression, and overall burden	Cross sectional study of 113 caregivers, 53 RT recipients and 60 caregivers of PD patients	51 (96.2%) of caregivers of RT recipients reported low CB and 30 (50%) of caregivers of patients on PD reported moderate CB using ZBI	CB scores were significantly higher in caregivers of PD patients compared with RT patients. The demographic data of caregivers did not significantly affect CB in either group including age, gender, income, and educational level. Duration of caregiving in year was not associated to CB.
Avsar et al. (2015) Turkey	To compare the caregivers of HD patients and caregivers of patients with RT in terms of anxiety, depression, sleep quality, and CB	Cross sectional study of 133 caregivers, 65 caregivers in the RT group and 68 in the HD group	62 (95.4%) of caregivers of RT patients reported low CB. 31 (45.6%) of caregivers of HD patients reported low CB and 27 (39.7%) of them reported moderate CB using ZBI	CB scores were significantly higher for caregivers in the HD group compared with caregivers in the RT group.
Bardak et al. (2018) Turkey	To compare CB, psychological symptoms in caregivers of PD, HD, and RT, and find out associated factors	Cross sectional study of 127 caregivers caring for 43 PD, 42 HD, 42 RT patients	Participants reported mild to moderate CB in all caregiver groups (PD, HD, RT) using ZBI.	CB score was found to be higher in caregivers who reported higher level of psychological symptoms (anxiety and depression). CB score was found to be highest in caregivers of patients receiving HD, and it was significantly higher than PD and RT groups. The gender, age, occupation, marital status, and education level of caregivers and patients were not associated with ZBI score. ZBI score was also not associated to caregiver relationships to the patient, living in the same house, or whether caregivers take all responsibility alone or not. Longer duration of HD affected the ZBI scores positively.
Bayoumi (2014) Saudi Arabia	To evaluate the CB of individuals who provide care for patients on maintenance HD	Cross sectional study of 50 caregivers for patients on HD	Moderate to severe burden using ZBI	The total caregiver burden significantly correlated positively with patient age and negatively correlated with caregiver age. Negative correlations were identified between caregivers’ and patients’ levels of education in relation to CB.
Belasco and Sesso (2002) Brazil	To describe the characteristics of caregivers of chronic HD patients, assess their perceived burden and HRQoL and the factors influencing this burden	Cross sectional study of 100 caregivers of HD patients	Moderate CB using CBS	Caregivers of male patients with a low education level (illiterate or primary), with a multiple number of patient comorbid conditions had a higher mean score burden. Caregivers who were female spouses of patients, and caring for long lengths of time, perceived a significantly greater burden than those with other types of relationships.
Belasco et al. (2006) Brazil	To describe caregivers’ characteristics and evaluate their burden and QoL	Cross sectional study of 201 caregivers, 161 caregivers of HD patients and 40 caregivers of PD patients	Moderate burden for both caregivers of elderly HD and PD patients using CBS	There is a significant difference noted in the environment dimension of CBS, which was better for caregivers of the elderly receiving HD than in younger patients. Caregiver’s mental status score was lower for caregivers of elderly receiving PD than caregivers of patient receiving HD. No significant influence of caregiver sex detected.
Cagan et al. (2018) Turkey	To examine the burden of caregivers of HD patients and some related variables	Cross sectional study of 163 caregivers of HD patients	Moderate CB using ZBI	Higher CB reported in female caregivers and in those who were being employed, having 3 or more children, having difficulty in meeting their health expenses (poor income), reporting that their role in the family and work is negatively affected, and giving care for longer than 5 years. There was no correlation between CB and caregivers’ age, marital status, educational status, place of residence (county, town, village, province), type of personality, smoking and alcohol consumption and number of weekly dialysis sessions.
Cantekin et al. (2016) Turkey	To determine the burden on primary caregivers of patients undergoing dialysis	Cross sectional survey of 114 patients, 54 were relatives of HD patients and 60 were relatives of PD patients	Caregivers of HD patients reported moderate to high burden while caregivers of PD reported low to medium levels of CB using ZBI	Caregivers of PD patients had lower levels of burden than caregivers of HD patients, and this was highly significant.
Faridah et al. (2020) Indonesia	To determine the factors associated with the CB of caring families of HD patients	Cross sectional survey of 95 caregivers of HD patients	No burden to low burden using ZBI	High CB is associated with the low salary and older age of caregivers. Duration of care (time), gender, and education level of caregivers were not significant in relation to CB.
Harris et al. (2000) USA	To identify the level of CB reported by African American caregivers of patients with ESKD waiting for RT and to identify whether subjective burdens varied by caregiver age	Cross sectional survey of 78 African American family caregivers of patients with ESKD	Little to no burden using ZBI	There was no significant difference in the level of subjective burden reported by young and older African-American caregivers.
Hoang et al. (2019) Vietnam	To analyse the burden and support activities of informal caregivers caring for adults receiving haemodialysis	Cross-sectional study recruited 178 adult informal caregivers of patients receiving HD	Moderate to severe burden using ZBI	Being old, married to the care recipients, having comorbidity condition, longer time spent on caregiving tasks, duration of being caregiver, and having difficulty of doing caregiving tasks were significantly associated with increasing the CB. Employment types of caregivers, income, and being a relative to the patients have no effect on CB
Jafari et al. (2018) Iran	To determine the level of CB and its relationship with the QoL of caregivers of HD patients	Cross sectional study of 246 caregivers of HD patients	42.7% experiencing moderate CB and 37.4% were experiencing high to severe levels of CB, using the Novak and Guest Care Burden Questionnaire	A significant positive correlation between the age of caregivers and CB. Increased education level decreased CB. With the increasing capability of patients in self-care, the CB of the caregiver decreased. High CB associated with the presence of comorbidity conditions. Significant and negative correlations between the total scores of CB and QoL. Patient age, caregiver and patient gender, occupation, education, and income were not associated with CB levels.
Joy et al. (2019) India	To assess the level of caregiver burden and resilience in caregivers of haemodialysis patients	Cross sectional study in 120 caregivers of patients on maintenance HD	Moderate to severe burden using ZBI	Duration of caregiving and time spent per day looking after the patients were associated positively with CB. Female caregivers reported higher burden than male caregivers. Age of caregivers was not associated with CB. Caregivers with low ability of adaptation to caregiving role had high burden.
Kang et al. (2019) Singapore	To examine changes in burden and QOL in caregivers of prevalent PD patients over 12 months	Longitudinal study in 44 caregivers of PD patients	Mild to moderate burden using ZBI	CB significantly increased over time. Over a 1 year period, the level of CB increased from mild to moderate burden to moderate to severe burden.
Kilic and Kaptanogullari (2017) Turkey	To evaluate the burden of caregivers who provided care to HD patients in two different communities	Cross sectional study of 210 caregivers of patients receiving HD	In Turkey (central district), mild CB & in Northern Cyprus (rural area) moderate CB, using ZBI	Caregiver to patient relationships: spouses had lower CB scores compared to other caregivers such as children, grandmothers/grandfathers, or siblings. Caregivers who live in the central district reported lower CB than those who live in rural areas. Caregiver age, sex, marital status, and educational status have no relation.
Mashayekhi et al. (2015) Iran	To assess the level of CB in caregivers of HD patients	Cross sectional study of 51 caregivers of HD patients	Moderate to severe levels of CB using CBS	Caregivers with inadequate income, caring for male patients had a higher CB score. Single caregivers gained higher scores of CB. No significant relationship between CB and patient’s occupation, education, marital status, comorbidities, duration of dialysis, level of patient dependency and frequency of HD per week. Education, occupation, gender, age of caregiver and kind of relationship with patient were associated to CB.
Mollaoglu et al. (2013) Turkey	To determine the burden, educational needs, influential factors, and the effects of home care education over CB among primary caregivers of patients undergoing HD	Pre and post-test design including 122 caregivers of HD patients	Moderate to severe, using ZBI	CB score was significantly higher in caregivers who are female, single than in married, young, caregivers with higher education and caregivers with comorbidity conditions compared to those who have no health problems. The spouses had a lower mean value of CB compared to those of daughters, daughters-in-law, and sisters/brothers Caregivers who live in a peripheral district or small town were found to have high CB, whereas those living in a central district of a province had low CB scores. Caregiver occupation and income were observed to have no impact on CB.
Nagarathnam et al. (2019) India	To evaluate the burden, coping mechanisms, and QOL among caregivers of HD and PD undergoing and RT patients	Cross sectional study of 90 patients (30 HD, 30 PD, and 30 RT patients)	Moderate to severe burden observed in caregivers of patients receiving HD, mild to moderate burden in patients receiving PD, and no burden were observed in RT patients, using ZBI	Significantly higher burden score in caregivers of HD undergoing than RT patients.
Oyegbile and Brysiewicz (2017) Nigeria	To explore the CB of family caregivers of ESKD patients in South-West Nigeria	Mixed method study including a cross sectional survey of 96 family caregivers of patients with ESKD	Moderate to severe CB using ZBI	Female caregivers experienced more burden of caregiving than their male counterparts. Caregivers living with patients experienced more burden of caregiving than those who live in separate residences. However, these did not reach statistically significant results. No significant differences in CB according to caregiver marital status, educational levels, religion, ethnicity, working status, relationship with patients, and duration of contact with patient.
Paschou et al. (2018) Greece	To explore the CB and depression in spouses of patients with CKD	Cross sectional study of 50 spouses of patients; 29 of whom were dialysis dependent and 21 were not dialysis dependent.	Moderate burden using ZBI	Caregiver age, gender, marital satisfaction, education, employment status and whether the patients were dialysis dependent or not have no relation to level CB. Higher levels of depression correlated to the increased perceived CB.
Rafati et al. (2019) Iran	To examine the relationship between caregiver burden and spiritual well-being in caregivers of patients receiving HD	Cross sectional-correlational study was conducted on 382 caregivers of patients receiving HD	Moderate to severe CB using the Novak and Guest Care Burden Questionnaire	A significant high CB was reported in caregivers who have a lower level of education, unemployed, lower spiritual well-being, older age. A significant high CB was reported in caregivers whose patients with low income, performing higher number of dialysis sessions per week, having lower level of dependency, and not having a kidney transplant history.
Rioux et al. (2012) Canada	To assess CB, QOL, and depressive symptoms and to compare these with their patients’	Cross sectional study of 61 caregivers of HD patients	Low level burden perceived using CBS	Depression was correlated positively to CB.
Rodrigue et al. (2010) USA	To characterise the psychosocial functioning of spouse/partner caregivers	Cross sectional study spouse/partner caregivers of HD and PD patients before (*n* = 33) and after (*n* = 46) kidney transplantation	High CB before and after transplantation using CSI	CB was not associated with age, sex, or relationship duration even though older females reported high CB but did not reach statistical significance. High levels of patient physical health associated with lower CB. CB was higher when the patient was on HD than not yet on dialysis. Higher CB was associated with worse patient health and lower mental QOL. No differences between CB of patients before and after RT.
Senmar et al. (2019) Iran	To measure CB among caregivers of older patients receiving HD and its relevant factors	Cross sectional study in 52 caregivers of elderly patients receiving HD	Moderate to severe level of CB using Novak and Guest Caregiver Burden questionnaire.	High age of caregivers, high age of patients and the caregiver-patient relationship were factors that significantly associated with increasing the level of CB. Gender of patients and caregivers, marital status of patients and caregivers, education level of caregivers, the income of patients and duration of dialysis were not associated with CB. Spouses had higher level of CB than other relationship.
Shah et al. (2017) Pakistan	To determine CB of patients receiving dialysis	Cross sectional study 164 caregivers of patients receiving dialysis	Mild to moderate CB using ZBI	A positive correlation was found between the duration of patient on dialysis/year, daily hours of caregiving and the total CB score. Low social class, high level of patient functional dependency associated with high CB.
Shakya et al. (2017) Nepal	To assess the burden on caregivers and find out their liability for developing depression	Cross sectional, descriptive study design. 164 caregivers of patients taking maintenance HD	The mean CB score was 46.99(14.6) indicating moderate to severe burden using ZBI	CB increased with increasing caregiver age, decreasing education, low socio-economic status and decreasing social support. Widow caregiver reported high CB then in married and the lowest CB was on single. Relationship to patient were also found to affect burden (spouses and parents having higher CB than in children, siblings, children in law, and grandchildren). CB was significantly positively associated with caregiver depression. However, duration of dialysis, frequency of dialysis, comorbid illness of (patients) were not found to have any significant association with CB.
Shimoyama et al. (2003) Japan	To examine the relationship in Japan between PD patients and caregivers with regard to HRQOL and CB	Cross sectional survey 34 caregivers of PD patients	Mean CB was 14.1 indicating little to No burden using ZBI	Caregivers of patient receiving PD reported low CB levels. CB associated with increasing age of patients and decreasing health-related QoL of caregivers.
Tao et al. (2020) China	To examine the level of CB on family caregivers of elderly adults receiving PD and to identify any contributing factors	Cross sectional survey 60 caregivers of PD patients	Mild to moderate CB using ZBI	Being female caregiver with insufficient financial status, low level of social support for the caregiver, depressive symptoms in the patients and caring for a patient with disability were statistically significant predictors of CB. Patient gender and educational level were not associated with CB. Caregiver educational level, living with the patients at the same resident, relationship with patients, and hours of caregiving per week was not associated with CB.
Washio et al. (2012) Japan	To investigate factors related to burden among caregivers of regular HD patients	Cross sectional survey 108 caregivers of HD	Mean CB score of 29 indicating mild burden to moderate using ZBI	Being spouse, having chronic diseases, and long time spent on caregiving is reported to be a related factor to the heavy burden among caregivers.
Wicks et al. (1997) USA	To explore QoL and CB reported by caregivers of persons with ESKD and to examine the relationship between these variables	Cross sectional design 96 caregivers of 96 RT candidates diagnosed with ESKD	Little to no burden using ZBI	Neither caregiver race, gender, relationship to the patient, caregiver health level, nor patient gender significantly contributed to CB. CB did not differ by dialysis type (PD, incenter HD, etc.) or employment category (full-time, part-time, etc.). Caregivers Qol related negatively with CB.
Zhang et al. (2016) China	To assess the burden for caring patients on maintenance HD by primary family caregivers	Cross sectional survey 151 caregiver of HD patients	151 caregivers, 51% of them reported mild to moderate burden and 25.2% caregivers reported moderate to severe burden using ZBI	Caregiver age increasing, low educational levels, without job, long caring hours were associated with high CB. CB was at lesser degree with high level of caregiver health. CB was significantly increased in patients with more than two comorbidities and patient’s low income. CB did not associate with duration of HD. Relationship with patients, spouses, parents, and adult children felt more stressful than siblings, daughters- and sons-in-law.
Zhang et al. (2020) China	To assess the burden in primary family members caring for uremic patients on PD	Cross sectional design on 170 PD patients	60% of caregivers reported mild to moderate burden and 18.2% reported moderate to severe burden using ZBI	Duration of PD and presence of comorbidity in patients, being female caregivers, spending longer hours providing care to patients were associated with higher CB. Caregivers who lived with patient had higher CB than those who lived separately. Caregivers who exercised for more than 1 h a week had a lower CB. CB is negatively associated with Qol. Age of caregivers, educational level, relationship to patients, and annual income were not associated with CB. Level of patients. Frailty was associated positively with CB.

HD = haemodialysis; CB = caregiver burden; CSA = caregiving stress appraisal; OCBS = Oberst Caregiving Burden Scale; PD = peritoneal dialysis; ZBI = Zarit Burden Interview; RT = renal transplant; HRQoL = health-related quality of life; CBS = Caregiver Burden Scale; QoL = quality of life; ESKD = end-stage kidney disease; CKD = chronic kidney disease; CSI = Caregiver Strain Index.

**Table 2:** Summarises the factors associated with CB.

	Abed et al.	Adejumo et al.	Affinito et al.	Alnazly	Alvarez et al.	Al Wakeel & Bayoumi	Avsar et al.	Avsar et al.	Bardak et al.	Bayoumi	Belasco & Sesso	Belasco et al.	Cagan et al.	Cantekin et al.	Faridah et al.	Harris et al.	Hoang et al.	Jafari et al.	Joy et al.	Kang et al.	Kilic & Kaptanogullari	Mashayekhi et al.	Mollaoğlu et al.	Nagarathnam et al.	Oyegbile	Paschou et al.	Rafati et al.	Rioux et al.	Rodrigue et al.	Senmar et al.	Shah et al.	Shakya et al.	Shimoyama et al.	Tao et al.	Washio et al.	Wicks et al.	Zhang et al.	Zhang et al.
Caregiver Age	s			n		s	n		n	s	n		n	n	s	n	s	s	n		n	n	s			n	s		n	s		s					s	n
Patients Age	n			n	s				n	s	n	s						n				n					n			s			s					n
Caregiver gender	n	s		n			n		n		s	n	s	n	n			n	s		n	n	s		n	n			n	n				s		n		s
Patient gender	n			n					n		s	n						n				s					n			n				n		n		n
Caregiver education	n			n		s	n		n	s	n		n	n	n			s			n	n	s		n	n	s			n		s		n			s	n
Patient education	n			n					n	s	s											n					s							n				
Caregiver occupation	n								n		n		s				n	n				n	n		n	n	s									n	s	
Patient occupation	n								n		n											n					s											
Marital status of caregiver	n								n				n				s	n			n	s	s		n	n	n			n		s						
Marital status of patient	n								n													n					n			n								
caregiver income	n						n				n		s	n	s		n	n				s	n				n							s				n
Patient income	n										n																s			n							s	
Socio-economic class caregiver											s																				s	s						
Caregiver ethnicity and race																									n											n		
Caregiver Religion/ spirituality																									n		s											
Caregiving hours/day, week				n	s						n				n		s		s						n						s			n			s	s
Length of caregiving (months, years)	n			n			n				s		s	n			s		s	s															s			
Duration of patient illness						n				n																												
Duration of dialysis	n								s		n		s					n				n					n			n	s	n						s
Caregiver relationship to patient	n			n					n		s		n				n				s	n	s		n		n			s		s		n	s	n	s	n
Duration of relationship																																						
Cohabiting status				s					n		n		n												n									n				s
Place of residence (town, village) (rural–urban)													n								s		s															
Patient functional dependency level					s													s				n					s		s		s			s				s
Caregiver poor physical and mental health			s																																	s		
Patient comorbidity history	n				s						s							s				n										n					s	s
Caregiver comorbidity history	s				s						n		n				s						s												s			
Smoking and alcohol consumptions													n																									
Number of medication of patients					s																																	
Number of caregiver children													s																									
Number (frequency) of dialysis session													n									n					s					n						
Social support				s	s																											s		s				
Coping ability to caregiving role				s															s																			
Positive role caregiver			s																																			
HRQol of caregiver/QoL		n																s											s				s			s		s
Carer Depression and anxiety		s							s																	s		s				s			s			
Patient Depression and anxiety																																		s				
Dialysis modalities (HD, PD)					n	n			s			n		s																						n		
Dialysis dependent or not																										n			s									
Before and after RT							s	s	s															s			s		n									
Caregiver health literacy	s																s																					
Caregiver performing exercise																																						s

HRQoL = health-related quality of life, HD = haemodialysis, PD = peritoneal dialysis, RT = renal transplant, *n* = factor explored but did not show a significant association to CB, s = factor explored and showed a significant association to CB.

## Data Availability

Not applicable.

## Appendix A

**Table A1:** Quality appraisal of the included studies using JBI.

Included Studies	Were the Criteria for Inclusion in the Sample Clearly Defined?	Were the Study Subjects and the Setting Described in Detail?	Was Exposure Measured in a Valid and Reliable Way?	Were Objective, Standard Criteria Used for Measurement of the Condition?	Were Confounding Factors Identified?	Were Strategies to Deal with Confounding Factors Stated?	Were the Outcomes Measured in a Valid and Reliable Way?	Was Appropriate Statistical Analysis Used?	Quality Assessment Scores
Abed et al. (2020)	Yes	Unclear	Unclear	Yes	Unclear	Unclear	Yes	Yes	Moderate
Adejumo et al. (2019)	Unclear	Unclear	Yes	Yes	Unclear	Unclear	Yes	Yes	Moderate
Affinito et al. (2018)	Yes	Unclear	Unclear	Yes	Unclear	Unclear	Yes	Yes	Moderate
Alnazly (2016)	Yes	Unclear	Yes	Yes	Yes	Yes	Yes	Yes	High
Alvarez-Ude et al. (2004)	Yes	Unclear	Yes	Yes	Yes	Yes	Yes	Yes	High
Al Wakeel & Bayoumi (2016)	Yes	Unclear	Yes	Yes	Unclear	Unclear	Yes	Yes	Moderate
Avsar et al. (2013)	Yes	Unclear	Yes	Unclear	Yes	Yes	Yes	Yes	High
Avsar et al. (2015)	Yes	Unclear	Unclear	Unclear	Yes	Yes	Yes	Yes	Moderate
Bardak et al. (2018)	Yes	Unclear	Yes	Yes	Unclear	Unclear	Yes	Yes	Moderate
Bayoumi (2014)	Yes	Unclear	Unclear	Yes	Yes	Yes	Yes	Yes	High
Belasco and Sesso (2002)	Yes	Unclear	Yes	Yes	Yes	Yes	Yes	Yes	High
Belasco et al. (2006)	Yes	Unclear	Yes	Yes	Yes	Yes	Yes	Yes	High
Cagan et al. (2018)	Unclear	Unclear	Yes	Unclear	Unclear	Yes	Yes	Yes	Moderate
Cantekin et al. (2016)	Unclear	Unclear	Unclear	Unclear	Yes	Yes	Yes	Yes	Moderate
Faridah et al. (2020)	Unclear	Unclear	yes	unclear	Unclear	Unclear	Yes	yes	Moderate
Harris et al. (2000)	Yes	Unclear	Unclear	Unclear	Yes	Yes	Yes	Yes	Moderate
Hoang et al. (2019)	Yes	Unclear	Yes	Yes	Yes	Yes	Yes	Yes	High
Jafari et al. (2018)	Yes	Unclear	Yes	Yes	Yes	Yes	Yes	Yes	High
Joy et al. (2019)	unclear	Unclear	Yes	yes	Unclear	unclear	yes	Yes	Moderate
Kang et al. (2019)	Unclear	Unclear	Yes	Unclear	Unclear	unclear	Yes	Yes	Moderate
Kilic and Kaptanogullari (2017)	Yes	Unclear	Yes	Yes	Unclear	Unclear	Yes	Yes	Moderate
Mashayekhi et al. (2015)	Yes	Unclear	Yes	Yes	Unclear	Unclear	Yes	Yes	Moderate
Molloaoglu et al. (2013)	Yes	Unclear	Yes	Yes	Yes	Unclear	Yes	Yes	High
Nagarathnam et al. (2019)	Unclear	Unclear	Unclear	Yes	Unclear	Unclear	Yes	Yes	Moderate
Oyegbile and Brysiewicz (2017)	Yes	Unclear	Yes	Unclear	Yes	Yes	Yes	Yes	High
Paschou et al. (2018)	Unclear	Unclear	Yes	Unclear	Unclear	Unclear	Yes	Yes	Moderate
Rafati et al. (2019)	Yes	Unclear	Yes	Yes	Yes	Unclear	Yes	Yes	High
Rioux et al. (2012)	Yes	Unclear	Yes	Unclear	Unclear	Unclear	Yes	Yes	Moderate
Rodrigue et al. (2010)	Yes	Unclear	Yes	Unclear	yes	Unclear	Yes	Yes	Moderate
Senmar et al. (2019)	Yes	Yes	Yes	Yes	Unclear	Unclear	Yes	Yes	High
Shah et al. (2017)	Unclear	Unclear	Yes	Unclear	Unclear	Unclear	Yes	Yes	Moderate
Shakya et al. (2017)	Yes	Unclear	Yes	Unclear	Unclear	Unclear	Yes	Yes	Moderate
Shimoyama et al. (2003)	Unclear	Unclear	Yes	Yes	Unclear	Unclear	Yes	Yes	Moderate
Tao et al. (2020)	Yes	Yes	Yes	Yes	Yes	Yes	Yes	Yes	High
Washio et al. (2012)	Unclear	Unclear	Yes	Unclear	Yes	Unclear	Yes	Yes	Moderate
Wicks et al. (1997)	Yes	Unclear	Yes	Unclear	Yes	Yes	yes	yes	High
Zhang et al. (2016)	Unclear	Unclear	Yes	Unclear	Unclear	Unclear	Yes	Yes	Moderate
Zhang et al. (2020)	Yes	Yes	Yes	Yes	Unclear	Unclear	Yes	Yes	High

## References

[1] Hill N.R., Fatoba S.T., Oke J.L., Hirst J.A., O’Callaghan C.A., Lasserson D.S., Hobbs F.R. (2016). Global prevalence of chronic kidney disease—A systematic review and meta-analysis. PLoS ONE.

[2] Hoang V.L., Green T., Bonner A. (2018). Informal caregivers’ experiences of caring for people receiving dialysis: A mixed-methods systematic review. J. Ren. Care.

[3] Etters L., Goodall D., Harrison B.E. (2008). Caregiver burden among dementia patient caregivers: A review of the literature. J. Am. Acad. Nurse Pract..

[4] Zarit S.H., Todd P.A., Zarit J.M. (1986). Subjective burden of husbands and wives as caregivers: A longitudinal study. Gerontologist.

[5] Rodrigue J.R., Dimitri N., Reed A., Antonellis T., Pavlakis M., Johnson S.R., Mandelbrot D.A. (2010). Spouse caregivers of kidney transplant patients: Quality of life and psychosocial outcomes. Prog. Transplant..

[6] Oyegbile Y.O., Brysiewicz P. (2017). Exploring caregiver burden experienced by family caregivers of patients with End-Stage Renal Disease in Nigeria. Int. J. Afr. Nurs. Sci..

[7] Chiao C.-Y., Wu H.-S., Hsiao C.-Y. (2015). Caregiver burden for informal caregivers of patients with dementia: A systematic review. Int. Nurs. Rev..

[8] Moher D., Liberati A., Tetzlaff J., Altman D.G. (2009). Preferred reporting items for systematic reviews and meta-analyses: The PRISMA statement. J. Clin. Epidemiol..

[9] Bayoumi M.M. (2014). Subjective burden on family carers of hemodialysis patients. Open J. Nephrol..

[10] Shakya D. (2017). Burden and Depression among Caregivers of Hemodialysis Patients. PMCOA Palliat. Med. Care Open Access.

[11] Cagan O., Unsal A., Celik N., Yilmaz A.T., Culha I., Eren H.K. (2018). Care Burden of Caregivers of Hemodialysis Patients and Related Factors. Int. J. Caring Sci..

[12] Zhang R., Cui X., Zhuang H., Xie W., Iv L., Liu Y. (2016). The burden for caring patients on maintenance hemodialysis is influenced by social and demographic factors. Gen. Med..

[13] Aromataris E., Munn Z. (2017). Joanna Briggs Institute Reviewer’s Manual.

[14] Popay J., Roberts H., Sowden A., Petticrew M., Arai L., Rodgers M., Britten N., Roen K., Duffy S. (2006). Guidance on the Conduct of Narrative Synthesis in Systematic Reviews: A Product from the ESRC Methods Programme Version 1.

[15] Ioannidis J.P., Patsopoulos N.A., Rothstein H.R. (2008). Reasons or excuses for avoiding meta-analysis in forest plots. BMJ.

[16] Abed M.A., Khalifeh A.H., Khalil A.A., Darawad M.W., Moser D.K. (2020). Functional health literacy and caregiving burden among family caregivers of patients with end-stage renal disease. Res. Nurs. Health.

[17] Adejumo O.A., Iyawe I.O., Akinbodewa A.A., Abolarin O.S., Alli E.O. (2019). Burden, psychological well-being and quality of life of caregivers of end stage renal disease patients. Ghana Med. J..

[18] Affinito J., Louie K. (2018). Positive Coping and Self-Assessed Levels of Health and Burden in Unpaid Caregivers of Patients with End Stage Renal Disease Receiving Hemodialysis Therapy. Nephrol. Nurs. J..

[19] Alnazly E.K. (2016). Burden and coping strategies among J ordanian caregivers of patients undergoing hemodialysis. Hemodial. Int..

[20] Alvarez-Ude F., Valdés C., Estébanez C., Rebollo P. (2004). Health-related quality of life of family caregivers of dialysis patients. J. Nephrol..

[21] Al Wakeel J.S., Bayoumi M.M. (2016). Caregiver burden among peritoneal dialysis and hemodialysis family in Saudi Arabia. Kuwait Med. J..

[22] Avsar U., Avsar U., Cansever Z., Set T., Cankaya E., Kaya A., Gozubuyuk H., Saatci F., Keles M. (2013). Psychological and emotional status, and caregiver burden in caregivers of patients with peritoneal dialysis compared with caregivers of patients with renal transplantation. Transplant. Proc..

[23] Avşar U., Avşar U.Z., Cansever Z., Yucel A., Cankaya E., Certez H., Keles M., Aydınlı B., Yucelf N. (2015). Caregiver burden, anxiety, depression, and sleep quality differences in caregivers of hemodialysis patients compared with renal transplant patients. Transplant. Proc..

[24] Bardak S., Demir S., Aslan E., Turgutalp K., Celikcan H.D., Dolarslan M.E., Kılıcarslan C., Karasu F., Gunes A.J., Kurt C. (2019). The other side of the coin in renal replacement therapies: The burden on caregivers. Int. Urol. Nephrol..

[25] Belasco A., Barbosa D., Bettencourt A.R., Diccini S., Sesso R. (2006). Quality of life of family caregivers of elderly patients on hemodialysis and peritoneal dialysis. Am. J. Kidney Dis..

[26] Belasco A.G., Sesso R. (2002). Burden and quality of life of caregivers for hemodialysis patients. Am. J. Kidney Dis..

[27] Cantekin I., Kavurmacı M., Tan M. (2016). An analysis of caregiver burden of patients with hemodialysis and peritoneal dialysis. Hemodial. Int..

[28] Faridah V.N., Nursalam N., Agustini N.L.P.I.B., Lestari T.P., Suratmi S., Juanita F., Aris A. (2020). Determinants of the Caregiver Burden of CKD Patients Undergoing Hemodialysis. Int. J. Psychosoc. Rehabil..

[29] Harris T.T., Thomas C.M., Wicks M.N., Faulkner M.S. (2000). Subjective burden in young and older African-American caregivers of patients with end stage renal disease awaiting transplant/Commentary and response. Nephrol. Nurs. J..

[30] Hoang V.L., Green T., Bonner A. (2019). Informal caregivers of people undergoing haemodialysis: Associations between activities and burden. J. Ren. Care.

[31] Jafari H., Ebrahimi A., Aghaei A., Khatony A. (2018). The relationship between care burden and quality of life in caregivers of hemodialysis patients. BMC Nephrol..

[32] Joy J., TJ H.K., Abraham P.M., Gopalakrishnan S. (2019). Burden and resilience in caregivers of patients on maintenance haemodialysis. Int. J. Res. Med. Sci..

[33] Kilic H.F., Kaptanogullari H. (2017). A Bicommunal Study: Burden of Caregivers of Hemodialysis Patients. Int. J. Caring Sci..

[34] Mashayekhi F., Pilevarzadeh M., Rafati F. (2015). The assessment of caregiver burden in caregivers of hemodialysis patients. Mater. Soc. Med..

[35] Nagarathnam M., Sivakumar V., Latheef S. (2019). Burden, coping mechanisms, and quality of life among caregivers of hemodialysis and peritoneal dialysis undergoing and renal transplant patients. Indian J. Psychiatry.

[36] Paschou A., Damigos D., Skapinakis P., Siamopoulos K. (2018). The Relationship between Burden and Depression in Spouses of Chronic Kidney Disease Patients. Depress. Res. Treat..

[37] Rafati F., Mashayekhi F., Dastyar N. (2019). Caregiver Burden and Spiritual Well-being in Caregivers of Hemodialysis Patients. J. Relig. Health.

[38] Rioux J.P., Narayanan R., Chan C.T. (2012). Caregiver burden among nocturnal home hemodialysis patients. Hemodial. Int..

[39] Senmar M., Rafiei H., Yousefi F., Razaghpoor A., Bokharaei M. (2019). Caregiver burden among family caregivers of older patients receiving hemodialysis and its relevant factors. J. Nephropharmacol..

[40] Shah H.B.U., Atif I., Rashid F., Babar M.W., Arshad F., Qamar W., Khan O.A., Qadir M.L. (2017). Assessment of caregiver burden of patients receiving dialysis treatment in Rawalpindi. J. Pak. Med. Assoc..

[41] Shimoyama S., Hirakawa O., Yahiro K., Mizumachi T., Schreiner A., Kakuma T. (2003). Health-related quality of life and caregiver burden among peritoneal dialysis patients and their family caregivers in Japan. Perit. Dial. Int..

[42] Tao X., Chow S.K.Y., Zhang H., Huang J., Gu A., Jin Y., He Y., Li N. (2020). Family caregiver’s burden and the social support for older patients undergoing peritoneal dialysis. J. Ren. Care.

[43] Washio M., Yoshida H., Ura N., Ohnishi H., Togashi N., Sakauchi F., Arai Y., Mori M., Shimamoto K. (2012). Burden among family caregivers of patients on chronic hemodialysis in northern Japan. Int. Med. J..

[44] Wicks M.N., Milstead E.J., Hathaway D.K., Cetingok M., Hickey J.V., Currier H. (1997). Subjective burden and quality of life in family caregivers of patients with end stage renal disease. ANNA J..

[45] Zhang R., Pu C., Cui X., Zhang N., Li X., Zheng F. (2020). Burden in primary family caregivers caring for uremic patients on maintenance peritoneal dialysis. Perit. Dial. Int..

[46] Mollaoğlu M., Kayataş M., Yürügen B. (2013). Effects on caregiver burden of education related to home care in patients undergoing hemodialysis. Hemodial. Int..

[47] Kang A., Yu Z., Foo M., Chan C.M., Griva K. (2019). Evaluating burden and quality of life among caregivers of patients receiving peritoneal dialysis. Perit. Dial. Int..

[48] Zarit S.H., Reever K.E., Bach-Peterson J. (1980). Relatives of the impaired elderly: Correlates of feelings of burden. Gerontologist.

[49] Elmståhl S., Malmberg B., Annerstedt L. (1996). Caregiver’s burden of patients 3 years after stroke assessed by a novel caregiver burden scale. Arch. Phys. Med. Rehabil..

[50] Novak M., Guest C. (1989). Application of a Multidimensional Caregiver Burden Inventory1. Gerontologist.

[51] Bakas T., Austin J.K., Jessup S.L., Williams L.S., Oberst M.T. (2004). Time and difficulty of tasks provided by family caregivers of stroke survivors. J. Neurosci. Nurs..

[52] Abe K. (2007). Reconsidering the Caregiving Stress Appraisal scale: Validation and examination of its association with items used for assessing long-term care insurance in Japan. Arch. Gerontol. Geriatr..

[53] Robinson B.C. (1983). Validation of a Caregiver Strain Index. J. Gerontol..

[54] Abbasi A., Rahmani H., Shariati A., Asayesh H., Ashraf Rezaee N., Mollaei E., Shoori Bidgoli A., Bathaei S. (2012). The relationship between caring burden and coping strategies in hemodialysis patients caregivers. J. Urmia Nurs. Midwifery Fac..

[55] Arechabala M., Catoni M.I., Palma E., Barrios S. (2011). Depression and self-perceived burden of care by hemodialysis patients and their caregivers. Rev. Panam. Salud Publica Pan Am. J. Public Health.

[56] Gülpak M., Kocaöz S. (2014). The care burden and the affecting factors of individuals receiving hemodialysis treatment. TAF Prev. Med. Bull..

[57] Talebi M., Mokhtari Lakeh N., Rezasoltani P. (2016). Caregiver Burden in Caregivers of RenalF Patients under Hemodialysis. J. Holist. Nurs. Midwifery.

[58] Teixidó-Planas J., Velasco L.T., Suárez N.A., Mas A.C. (2018). Carer’s burden of peritoneal dialysis patients. Questionnaire and scale validation. Nefrol. Engl. Ed..

[59] Dastyar N., Mashayekhi F., Rafati F. (2020). Caregiving burden in hemodialysis patients’ caregivers in Kerman Province: A descriptive-analytical study. J. Jiroft Univ. Med Sci..

[60] Al-Rawashdeh S.Y., Lennie T.A., Chung M.L. (2016). Psychometrics of the Zarit Burden Interview in caregivers of patients with heart failure. J. Cardiovasc. Nurs..

[61] Chou K.R. (2000). Caregiver burden: A concept analysis. J. Pediatr. Nurs..

[62] Xiong C., Biscardi M., Astell A., Nalder E., Cameron J.I., Mihailidis A., Colantonio A. (2020). Sex and gender differences in caregiving burden experienced by family caregivers of persons with dementia: A systematic review. PLoS ONE.

[63] Chappell N.L., Dujela C., Smith A. (2015). Caregiver Well-Being:Intersections of Relationship and Gender. Res. Aging.

[64] Akpınar B., Küçükgüçlü Ö., Yener G. (2011). Effects of Gender on Burden Among Caregivers of Alzheimer’s Patients. J. Nurs. Scholarsh..

[65] Chaplin T.M. (2015). Gender and Emotion Expression: A Developmental Contextual Perspective. Emot. Rev..

[66] Kring A.M., Gordon A.H. (1998). Sex Differences in Emotion: Expression, Experience, and Physiology. J. Pers. Soc. Psychol..

[67] Almutary H., Bonner A., Douglas C. (2016). Which patients with chronic kidney disease have the greatest symptom burden? A comparative study of advanced ckd stage and dialysis modality. J. Ren. Care.

[68] Andrén S., Elmståhl S. (2007). Relationships between income, subjective health and caregiver burden in caregivers of people with dementia in group living care: A cross-sectional community-based study. Int. J. Nurs. Stud..

[69] Serrano-Aguilar P., Lopez-Bastida J., Yanes-Lopez V. (2006). Impact on health-related quality of life and perceived burden of informal caregivers of individuals with Alzheimer’s disease. Neuroepidemiology.

[70] Conde-Sala J.L., Garre-Olmo J., Turró-Garriga O., Vilalta-Franch J., López-Pousa S. (2010). Differential features of burden between spouse and adult-child caregivers of patients with Alzheimer’s disease: An exploratory comparative design. Int. J. Nurs. Stud..

[71] Ganapathy V., Graham G.D., DiBonaventura M.D., Gillard P.J., Goren A., Zorowitz R.D. (2015). Caregiver burden, productivity loss, and indirect costs associated with caring for patients with poststroke spasticity. Clin. Interv. Aging.

[72] Miller B., Montgomery A. (1990). Family caregivers and limitations in social activities. Res. Aging.

[73] Raccichini A., Spazzafumo L., Castellani S., Civerchia P., Pelliccioni G., Scarpino O. (2015). Living with mild to moderate Alzheimer patients increases the caregiver’s burden at 6 months. Am. J. Alzheimers Dis. Dement..

[74] Viñas-Diez V., Turró-Garriga O., Portellano-Ortiz C., Gascón-Bayarri J., Reñé-Ramírez R., Garre-Olmo J., Conde-Sala J.L. (2017). Kinship and cohabitation in relation to caregiver burden in the context of Alzheimer’s disease: A 24-month longitudinal study. Int. J. Geriatr. Psychiatry.

[75] Jung H.-Y., Jeon Y., Park Y., Kim Y.S., Kang S.-W., Yang C.W., Kim N.-H., Choi J.-Y., Cho J.-H., Park S.-H. (2019). Better quality of life of peritoneal dialysis compared to hemodialysis over a two-year period after dialysis initiation. Sci. Rep..

[76] Liu H., Fang B., Chan J., Chen G. (2019). The relationship between comorbidities in dementia patients and burden on adult–child primary caregivers: Does having a secondary caregiver matter?. Int. J. Ment. Health Nurs..

[77] Garcia R.S., Pinheiro B.V., Lucinda L.M., Pimentel A.L., Júnior J.M., Paula R.B., Reboredo M.M. (2020). Association between exercise training in haemodialysis patients and burden of their family caregivers: A cross-sectional study. Nephrology.

[78] Kim D. (2017). Relationships between Caregiving Stress, Depression, and Self-Esteem in Family Caregivers of Adults with a Disability. Occup. Ther. Int..

[79] Kim H., Chang M., Rose K., Kim S. (2012). Predictors of caregiver burden in caregivers of individuals with dementia. J. Adv. Nurs..

[80] Anngela-Cole L., Hilton J.M. (2009). The Role of Attitudes and Culture in Family Caregiving for Older Adults. Home Health Care Serv. Q..

[81] Yeh Y.J. (2006). Knowledge management enablers: A case study. Ind. Manag. Data Syst..

[82] Creedle C., Leak A., Deal A.M., Walton A.M., Talbert G., Riff B., Hornback A. (2012). The impact of education on caregiver burden on two inpatient oncology units. J. Cancer Educ..

[83] Burnley C.S. (1987). Caregiving: The impact on emotional support for single women. J. Aging Stud..

[84] Vaingankar J.A., Chong S.A., Abdin E., Picco L., Jeyagurunathan A., Zhang Y., Sambasivam R., Chua B.Y., Ng L.L., Prince M. (2016). Care participation and burden among informal caregivers of older adults with care needs and associations with dementia. Int. Psychogeriatr..

[85] Randall A.K., Bodenmann G. (2009). The role of stress on close relationships and marital satisfaction. Clin. Psychol. Rev..

[86] Lin I.F., Fee H.R., Wu H.S. (2012). Negative and Positive Caregiving Experiences: A Closer Look at the Intersection of Gender and Relatioships. Fam. Relat..

[87] Friedemann M.-L., Buckwalter K.C. (2014). Family Caregiver Role and Burden Related to Gender and Family Relationships. J. Fam. Nurs..

[88] Zeng L., Zhu X., Meng X., Mao Y., Wu Q., Shi Y., Zhou L. (2014). Responsibility and burden from the perspective of seniors’ family caregivers: A qualitative study in Shanghai, China. Int. J. Clin. Exp. Med..

[89] Arun R., Inbakamal S., Tharyan A., Premkumar P.S. (2018). Spousal caregiver burden and its relation with disability in schizophrenia. Indian J. Psychol. Med..

